# Chronic blue light-emitting diode exposure harvests gut dysbiosis related to cholesterol dysregulation

**DOI:** 10.3389/fcimb.2023.1320713

**Published:** 2024-01-08

**Authors:** Cheng-Hsieh Huang, Sebastian Yu, Hsu-Sheng Yu, Hung-Pin Tu, Yao-Tsung Yeh, Hsin-Su Yu

**Affiliations:** ^1^ Ph. D. Program in Environmental and Occupational Medicine, College of Medicine, Kaohsiung Medical University and National Health Research Institutes, Kaohsiung, Taiwan; ^2^ Aging and Disease Prevention Research Center, Fooyin University, Kaohsiung, Taiwan; ^3^ Department of Dermatology, Kaohsiung Medical University Hospital, Kaohsiung Medical University, Kaohsiung, Taiwan; ^4^ Department of Food Science, National Pingtung University of Science and Technology, Pingtung, Taiwan; ^5^ Department of Public Health and Environmental Medicine, School of Medicine, College of Medicine, Kaohsiung Medical University, Kaohsiung, Taiwan; ^6^ Department of Medical Laboratory Sciences and Biotechnology, Fooyin University, Kaohsiung, Taiwan; ^7^ National Institute of Environmental Health Sciences, National Health Research Institutes, Miaoli, Taiwan; ^8^ Graduate Institute of Clinical Medicine, College of Medicine, Kaohsiung Medical University, Kaohsiung, Taiwan

**Keywords:** gut microbiota, cholesterol metabolism, gut dysbiosis, circadian disruption, light-emitting diode

## Abstract

Night shift workers have been associated with circadian dysregulation and metabolic disorders, which are tightly coevolved with gut microbiota. The chronic impacts of light-emitting diode (LED) lighting at night on gut microbiota and serum lipids were investigated. Male C57BL/6 mice were exposed to blue or white LED lighting at Zeitgeber time 13.5-14 (ZT; ZT0 is the onset of “lights on” and ZT12 is the “lights off” onset under 12-hour light, 12-hour dark schedule). After 33 weeks, only the high irradiance (7.2 J/cm^2^) of blue LED light reduced the alpha diversity of gut microbiota. The high irradiance of white LED light and the low irradiance (3.6 J/cm^2^) of both lights did not change microbial alpha diversity. However, the low irradiance, but not the high one, of both blue and white LED illuminations significantly increased serum total cholesterol (TCHO), but not triglyceride (TG). There was no significant difference of microbial abundance between two lights. The ratio of beneficial to harmful bacteria decreased at a low irradiance but increased at a high irradiance of blue light. Notably, this ratio was negatively correlated with serum TCHO but positively correlated with bile acid biosynthesis pathway. Therefore, chronic blue LED lighting at a high irradiance may harvest gut dysbiosis in association with decreased alpha diversity and the ratio of beneficial to harmful bacteria to specifically dysregulates TCHO metabolism in mice. Night shift workers are recommended to be avoid of blue LED lighting for a long and lasting time.

## Introduction

Circadian rhythm (CR) refers to an endogenous process that regulates the repeated timing of physiological function (Green et al., 2008; Asher and Schibler, 2011; Eckel-Mahan and Sassone-Corsi, 2013), and also ensures that one’s internal physiology is synchronized with external environment (Gachon et al., 2004). In mammals, the suprachiasmatic nucleus (SCN) is the “master clock” responsible for coordinating and synchronizing daily rhythm via transmitting signals to other clocks in peripheral organs (Asher and Sassone-Corsi, 2015). External environmental cues such as light can train the CR to be synchronized with the light–dark (LD) cycle through the circadian clock (Mukherji et al., 2013; Thaiss et al., 2016; Wang et al., 2017). Light information can be transmitted by the retino-hypothalamic tract linking the eye to the SCN (Panda et al., 2002). The non-visual effects of light such as CR regulation are mediated by a retinal photoreceptor system built from the intrinsically photosensitive retinal ganglion cells (ipRGC) (Zaidi et al., 2007; Guler et al., 2008). In these unique ipRGC cells, the triggering signal transduction is accomplished by the photopigment melanopsin, which shows maximum sensitivity to the blue, i.e., short wavelength, part of the spectrum (∼480 nm) (Berson et al., 2002; Hattar et al., 2002). Although the genes involved in maintaining the CR drive many biological pathways, they are particularly relevant in metabolism (Sancar and Brunner, 2014; Mayeuf-Louchart et al., 2017). Therefore, following CR disruption, the metabolism and energy networks consequently become imbalanced, resulting in disorders such as obesity (Rudic et al., 2004; Evans and Davidson, 2013). CR can also regulate hepatic lipid metabolism and thus the disruption of CR can further result in the dysregulation of serum lipid (Wang and Liangpunsakul, 2016). It is important to note that abnormal TCHO rather than TG metabolism is suggested to be a critical event in the pathogenesis of metabolic syndrome (Swinnen and Verhoeven, 1998; Lee et al., 2003), and hypercholesterolemia seems to be one of the most important risk factors involved in cardiovascular disease (CVD), coronary heart disease, and type 2 diabetes (Besseling et al., 2015; Cimminiello et al., 2016).

Light-emitting diodes (LEDs) are widely used in electronic devices and are recognized as a major source of circadian dysregulation in modern society. The overuse of these devices has been increasingly linked to sedentary behavior at home (Granich et al., 2011), which presents a cardio-metabolic risk (Owen et al., 2010). White LED light is the most used lighting source worldwide and contains a harmful blue band (400-500 nm) (Touitou and Point, 2020) and about 20~30% blue light and 15~18% red light (Kevin R Cope, 2013). Although the light emitted by most of LEDs appears to be white, LEDs have a peak emission in the blue color range (Tosini et al., 2016). In humans, circadian responses to light are most sensitive to blue light (Brainard et al., 2001; Thapan et al., 2001; Wright and Lack, 2001; Lockley et al., 2003). Men show a strong response to blue light in the late evening, even at very low levels, compared to women (Chellappa et al., 2017b). Artificial light at night (ALAN) is associated with a higher risk of CVD (Morris et al., 2016; Chellappa et al., 2017a). Most studies have indicated the effects of ALAN on the disruption of metabolic processes, resulting in obesity or diabetes and cancer incidence (Russart and Nelson, 2018). Nightshift work is also associated with a range of adverse health outcomes such as metabolic diseases (Kiley and Fahy, 2018). Interestingly, on the other hand, blue light can be filtered out in electronic screens during the night (Driller et al., 2019; Teran et al., 2020), and blue-enriched light is used by nightshift workers to optimize their body rhythm for achieving maximum performance (Motamedzadeh et al., 2017; Sletten et al., 2017; Sletten et al., 2021). In addition, the long-term reduction of short-wavelength light reduces sustained attention and visuospatial working memory but there is no evidence that it causes a change in circadian rhythmicity (Domagalik et al., 2020). The chronic impacts of blue LED lighting on health as well as the metabolism remain to be investigated.

Mounting evidence has revealed that the gut microbiota, the circadian clock, and the metabolic system are tightly co-evolved (Mukherji et al., 2013; Thaiss et al., 2014; Leone et al., 2015; Thaiss et al., 2016; Wang et al., 2017). Although gut microbes are not directly exposed to light, diurnal host signals can induce oscillations in both the abundance and function of gut bacteria (Zarrinpar et al., 2014; Leone et al., 2015; Liang et al., 2015). Emerging research has also demonstrated that gut microbes are capable of programming the host’s energy balance, such as lipid absorption and storage, and have a major impact on metabolic homeostasis (Turnbaugh et al., 2006; Martinez-Guryn et al., 2018). Moreover, gut microbiota are very sensitive to environmental changes, including dietary and physiological, changes, and exhibit rapid responses in both community members and functions, effects that can quickly feedback onto the host’s health outcomes (Devkota et al., 2012; Huang et al., 2013; David et al., 2014). Gut dysbiosis is a health outcome in which biodiversity is strongly reduced and the microbial action becomes progressively pro-inflammatory and detrimental to human health (Hooks and O'Malley, 2017; Brussow, 2020). In contrast, eubiosis is characterized by a high biodiversity, a harmonic inter-microbial condition and mutualistic relationship between the microbiota and the host. A eubiotic state can be recognized as having a “balanced” between beneficial and harmful bacteria (Lepage et al., 2013; Iebba et al., 2016). An increased ratio of beneficial to harmful bacteria can improve gut dysbiosis-related disorders, even with the administration of probiotics such as *Lactobacillus* species (Khare and Gaur, 2020). Similar probiotic approaches could be used to correct, re-establish, or maintain appropriate rhythm in the gut flora population. Animal studies have suggested that circadian disruption, similar to that in shift workers, contributes to the development of gastrointestinal complaints among shift workers by altering the composition and normal diurnal rhythmicity of the resident intestinal microbes (De Bacquer et al., 2009). Although some studies have pointed out no differences in the composition of the fecal microbiome between lean and obese individuals (Ley et al., 2006; Turnbaugh et al., 2009), other studies in humans show that *Firmicutes* are associated with a higher propensity for obesity and metabolic disease (Ley et al., 2006; Finucane et al., 2014), conditions that are more common in night and rotating shift workers (Suwazono et al., 2008; De Bacquer et al., 2009). The environmental disruption of mouse and human CR by phase-shifting LD cycles and jet lag across time zones, respectively, significantly altered 24-hour bacterial patterning. It can be proposed that the chronic exposure to LEDs’ illumination might disrupt CR and associated gut microbiota, harvesting a diseased niche in the gut and changing metabolic homeostasis. However, the information about long-lasting/chronic effects of artificial light (in particular a specific wavelength of LEDs) on the gut microbiota and metabolic changes is limited.

In the present study, we employed a mouse model to ascertain the chronic effects of blue and white LED lighting with a low and high irradiance on the gut microbiota and associated serum lipids. We found chronic blue LED lighting in particular decreased microbial α diversity and modulated the abundances and ratio of a specific set of gut microbes, causing increased serum TCHO, but not TG, in mice. Our results revealed that chronic blue LED lighting would harvest gut dysbiosis in relation to TCHO dysregulation through the disruption of CR and bile acid biosynthesis.

## Results

### Chronic blue LED light exposure reduced the α-diversity of gut microbiota

**Figure 1:**
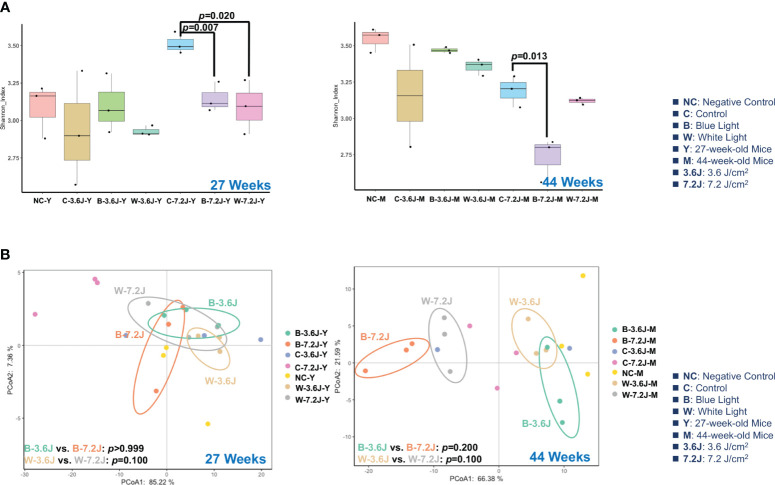
Chronic LED lighting resulted in gut dysbiosis especially when exposed to blue LED lighting. The gut microbiota composition was analyzed by 16S rRNA amplicon sequencing of the C57BL/6 mice after 27 and 44 weeks of exposure to blue and white LED lighting. **(A)** The Shannon diversity index (mean ± SEM) of the microbial community of the control group and the groups exposed to chronic LED lighting after 27 and 44 weeks. **(B)** A PCoA plot showing the beta diversity of the gut microbiome community at the OTU level of 27 and 44 weeks with a weighted UniFrac distance derived from 16S rRNA sequencing data. There was no significance between white and blue LED lighting. SEM: the standard error of the mean. The Shannon diversity index was analyzed by an unpaired Student’s t-test. *p*<0.05 was considered statistically significant. A one-way PERMANOVA test was shown for the beta diversity analysis (*p* values).

To explore the impacts of the chronic irradiance of blue and white LED light, mice were set under a dark phase and randomly categorized into four groups, including a negative control group without a cage shift and LED lighting, and experimental groups receiving two regular and fixed exposure times of blue and white LED lighting from 11 weeks to 44 weeks. A long exposure time/high irradiance (7.2 J/cm^2^) of blue LED light significantly decreased the α-diversity of fecal microbiota as compared with the matched control group at both 27 and 44 weeks (*p*=0.007 and 0.013, respectively; **Figure 1A**). Chronic white LED lighting decreased the microbial α-diversity only at high irradiance and at 27 weeks (p=0.020, **Figure 1A**). It is noted that the lower α-diversity of the gut microbiota is associated with several diseases including obesity, diabetes, and colorectal cancer (Mosca et al., 2016; Kriss et al., 2018; Prehn-Kristensen et al., 2018).

The β-diversity of the microbial compositions was not significantly different between each group when using Principal Coordinate Analysis (PCoA) with the weighted UniFrac distance method (**Figure 1B**). However, it is noted that the microbial compositions of each group were obviously separated at 44 weeks (**Figure 1B**). These results implied that high irradiance and a long time period were required for the LED lighting-mediated compositional change in the gut microbes. In addition, chronic exposure to the high irradiance of blue LED lighting decreased the α-diversity of the gut microbiota, suggesting that chronic blue LED lighting might harvest a diseased niche such as gut dysbiosis.

### Chronic LED lighting mainly changed the abundance of lipid metabolism-related gut microbes

**Figure 2:**
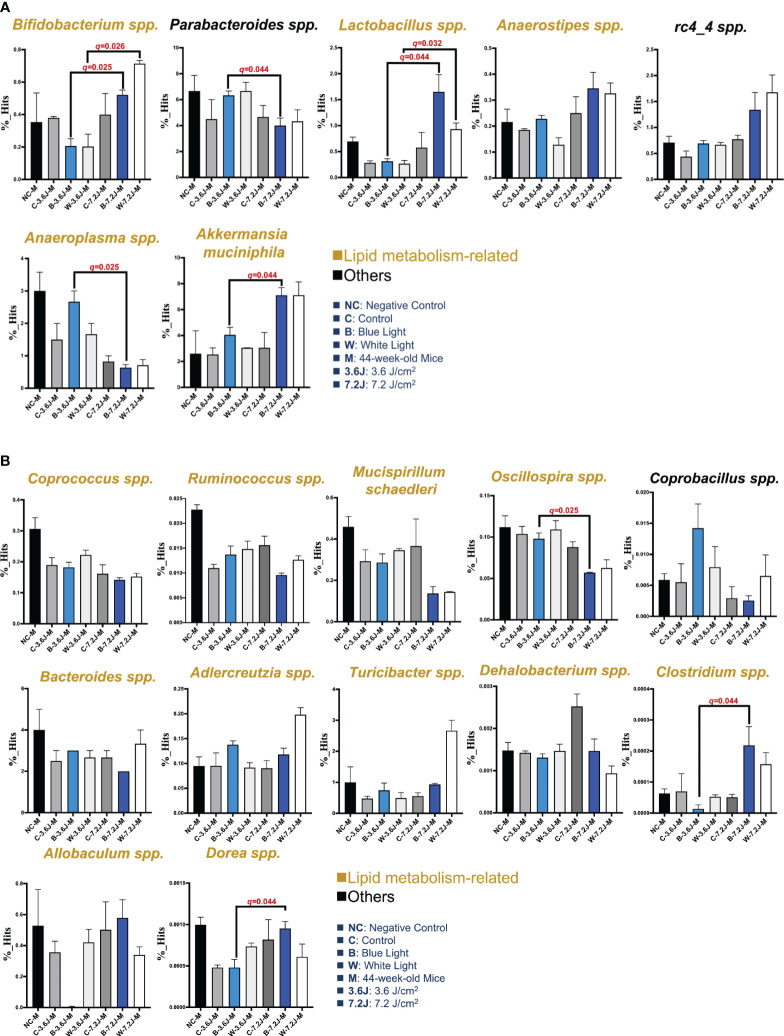
Core microbiota changed after chronic LED lighting. **(A)** The bar plot of the bacteria deduced from both the LEfSe analysis after 44 weeks of exposure to LED lighting. **(B)** The bar plot of the bacteria deduced by the heatmap analysis after 44 weeks of exposure to LED lighting. Lipid metabolism-related bacteria comparable to level 2 (i.e., KEGG) were presented in the color yellow. Others (i.e., other mechanisms in addition to lipid-related ones) were presented in black. All the results were also checked by the Benjamini-Hochberg false discovery rate (FDR) method. A *q*-value less than 0.05 was considered statistically significant.

Circadian dysregulation has been regarded as one of the risk factors for a wide spectrum of diseases such as metabolic syndrome (Bass and Takahashi, 2010). Circadian disruption and consequent dysregulation by chronic blue LED lighting might be associated with gut dysbiosis as shown above (**Figure 1**). The LEfSe and heatmap abundance analysis were used to explore the critical bacteria changed by chronic LED lighting (**Supplementary Figure 1**). It was noted that the low exposure time of both types of chronic LED lighting could not make significant changes in the microbial compositions at 27 weeks (data not shown). Intriguingly, 15 to 19 bacteria detected from LEfSe and heatmaps were related to lipid metabolism according to the Kyoto Encyclopedia of Genes and Genomes (KEGG), level 2 (16/19, in yellow) and other pathways (3/19, in black) (**Figure 2**). Interestingly, after the high irradiance of blue LED light at 44 weeks, compared to the matched control, *Akkermansia muciniphila*, well-known third generation probiotics (Zhang et al., 2019), were increased (*p=*0.037 and *q*=0.044), **Figure 2A**), while *Oscillospira* spp. were found to be reduced at a high level of irradiance of blue LED light (*p*=0.031 and *q*=0.025, **Figure 2B**). *Clostridium* spp. could also be induced (*p=*0.049 and *q*=0.044, **Figure 2B**). A high irradiance of both types of LED lighting could signify that chronic LED lighting would change the composition of gut microbiota by specific wavelengths of LED lighting.

These critical bacteria altered by chronic LED lighting might be merely a reflection of the aging process and other experimental factors such as cage shift. Therefore, in order to adjust the potential impacts of aging and the movement of the cage, the composition of gut microbiota at 11, 27 and 44 weeks of the negative control (NC), control (i.e., the cage shift without LED lighting on), and various LED lighting treatment group were rearranged and compared in **Supplementary Figure 2**. *Ruminococcus* spp. and *Bacteroides* spp. increased as the weeks increased (*p*<0.001 and 0.008, respectively; **Supplementary Figure 2**). Both low and high irradiances of blue and white LED lighting on *Bacteroides* spp. were similar to those seen in the negative control. The abundances of *Oscillospira* spp. and *Rumincoccus* spp. were both reduced in the group with high irradiance of blue LED lighting along with increasing age/exposure time (*p*=0.002 and 0.001, respectively; **Supplementary Figure 2**). In contrast, *Allobaculum* spp. were increased in the group with high irradiance of blue LED lighting along with aging (*p*=0.003, **Supplementary Figure 2**). In short, both types of LED lighting harvested gut dysbiosis with altered abundances of some overlapping and other distinct specific bacteria. However, the change in the core bacteria by chronic LED lighting was not a result of the aging and cage shift in our study, because there was an increasing trend in *Turicibacter* spp. (*p*=0.028 and 0.004, **Figure 2B**). *Dorea* spp. were the only bacteria-induced at low irradiance of white LED light (*p*=0.021, **Figure 2B**). These findings on chronic LED lighting were not in alignment with the trends related to aging.

### Chronic blue LED lighting-induced gut dysbiosis resulted in alterations in serum cholesterol but not triglyceride

To further examine the impacts of chronic blue and white LED lighting on metabolic homeostasis, the blood of each mouse was collected after 183 times of LED light exposure at 44 weeks. Intriguingly, chronic LED lighting at low irradiance, but not at high irradiance, caused impacts specifically on serum TCHO but not TG or glucose levels in mice. The low irradiance of both chronic blue and white LED lighting increased TCHO but not TG levels (*p*=0.029 and 0.033, respectively; **Table 1**).

**Table 1:** Blood biochemistry in the mice exposed to various irradiances of LED lighting after 44 weeks.

Group (J/cm^2^)	TCHO (mg/dL)	TG (mg/dL)	Glucose (mg/dL)
**C-3.6**	85.5 ± 1.5	39.0 ± 15.0	175.0 ± 24.0
**B-3.6**	95.9* ± 2.3	49.8 ± 13.4	172.8 ± 25.5
**W-3.6**	119.0* ± 1.5	64.0 ± 9.5	199.0 ± 26.7
**C-7.2**	87.7 ± 3.5	60.3 ± 9.4	179.3 ± 7.4
**B-7.2**	86.7 ± 5.6	67.9 ± 2.7	202.8 ± 17.8
**W-7.2**	82.5 ± 10.9	56.6 ± 13.5	267.5 ± 43.7
**NC**	98.7 ± 4.4	54.5 ± 12.0	256.4 ± 10.5

* Indicated p<0.05 as compared to C-3.6 J. Mean ± SEM.

### Chronic blue LED lighting changed the ratio of beneficial to harmful bacteria in a specific set, altering serum TCHO levels

**Figure 3:**
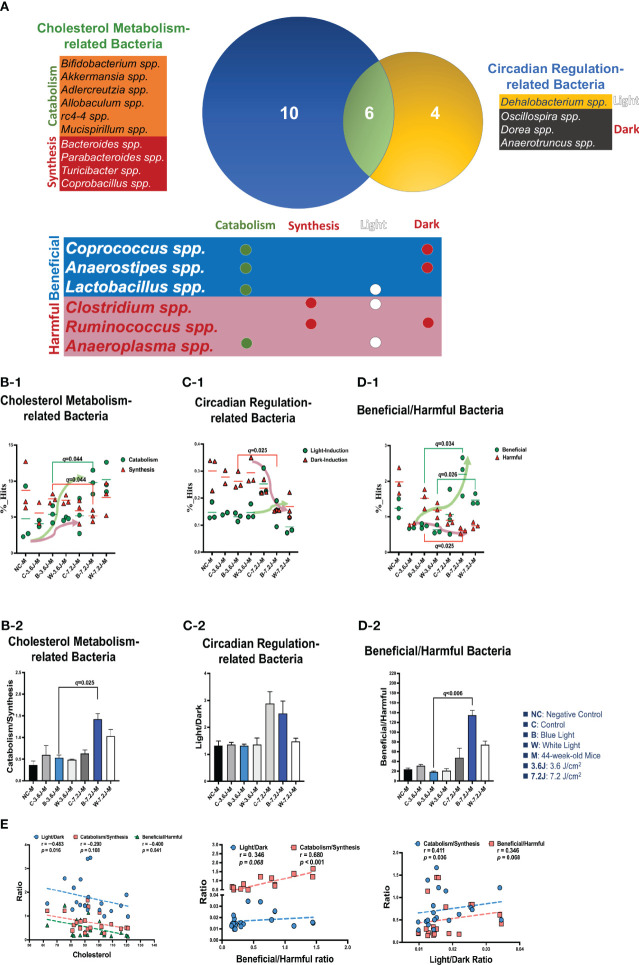
Chronic blue LED lighting altered the ratio of specific sets of TCHO metabolism-related bacteria and the ratio of beneficial to harmful bacteria. **(A)** A Venn plot of TCHO metabolism- and circadian regulation-related gut bacteria upon exposure to chronic LED lighting. Ten bacteria in a blue circle denoted as TCHO metabolism-related bacteria (in green color); six bacteria were related to TCHO catabolism (Zhang et al., 2016; Zietak et al., 2016; Kriaa et al., 2019; Vourakis et al., 2021), and four bacteria were in relation to TCHO synthesis (Granado-Serrano et al., 2019; Bourgin et al., 2020; Vourakis et al., 2021; Le et al., 2022). Four bacteria in a yellow circle were regarded as circadian regulation-related bacteria (in blue color), and one bacterium were induced by the dark (Kaczmarek et al., 2017). Six bacteria in a green circle were involved in both TCHO metabolism (Brown and Hazen, 2018) and two bacteria were involved in TCHO synthesis (Kenny et al., 2020) and circadian regulation (Lordan et al., 2020; Dempsey and Corr, 2022) were defined as beneficial bacteria (in blue color), and the other three were frequently regarded as harmful bacteria (in red color). There were six genera which were intersected between TCHO metabolism and circadian regulation. Ten bacteria were independently involved in TCHO metabolism, including catabolism and synthesis. Four bacteria were independently regulated by circadian rhythms, including light and dark phases. **(B-1, B-2)** The bar plot showed the ratio of catabolism-to-synthesis of TCHO metabolism-related bacteria. A high irradiance of blue LED lighting increased catabolism ability (*p*=0.011 and *q*=0.025)). The dot plot showed the changes of catabolism (green dot)/synthesis (red triangle)-related bacteria after chronic LED lighting. **(C-1, C-2)** The bar plot showed the ratio of light-induction to dark-induction of circadian-regulated bacteria. The dot plot showed the altered abundance of light-induced (green dot)/harmful (red triangle) bacteria after chronic LED lighting. **(D-1, D-2)** The bar plot showed the ratio of beneficial to harmful bacteria which were involved in both circadian regulation and TCHO metabolism. The dot plot showed the altered abundance of beneficial (green dot)/dark-induced (red triangle) bacteria after chronic LED lighting. **(E)** The ratio of beneficial to harmful bacteria is shown in red and was negatively correlated with serum TCHO (left). The ratio of beneficial to harmful bacteria was positively correlated with that of TCHO catabolism to synthesis (middle). The ratio of light to dark-induced bacteria was also positively correlated with that of TCHO catabolism to synthesis (right). The dot plot showed the abundance of beneficial (green dot)/harmful (red triangle) bacteria, respectively, after chronic LED lighting. All the results were also checked by Benjamini-Hochberg false discovery rate (FDR) method. A *q-*value less than 0.05 was considered statistically significant.

To explore why a low, but not a high, irradiance of chronic LED lighting altered the levels of blood lipid (**Figure 3A**), the Venn diagram plot was used and showed that four genera (*Dehalobacterium, Oscillospira, Dorea*, and *Anaerotruncus* spp. in the yellow circle, **Figure 3A**) were independently involved in circadian regulation and ten genera (*Bifidobacterium, Akkermansia, Adlercreutzia, Allobaculum, rc4-4, Mucispirillum, Bacteroides, Parabacteroides, Turicibacter* and *Copronacillus* spp. in the blue circle, **Figure 3A**) were independently included in TCHO metabolism. There were six genera (*Coprococcus, Anaerostipes, Lactobacillus, Clostridium, Ruminococcus*, and *Anaeroplasma* spp. in the green circle, **Figure 3A**) intersected in both circadian regulation- and TCHO metabolism-related gut bacteria (**Figure 3A**) (Brown and Hazen, 2018). To see the dynamic balance between the catabolism and biosynthesis of TCHO metabolism as well as between light and dark phase/induced CR regulation, the ratio of catabolism and biosynthesis was specifically analyzed in TCHO metabolism-related bacteria between groups (**Figure 3B-2**), and the ratio of light and dark induction was specifically analyzed in CR regulation-related bacteria between groups (**Figure 3B-2**). The ratio of catabolism-related bacteria (i.e., *Bifidobacterium*, *Akkermansia*, *Adlercreutzia*, *Allobaculum*, *rc4-4*, and *Mucispirillum* spp.) (Zhang et al., 2016; Zietak et al., 2016; Brown and Hazen, 2018; Kriaa et al., 2019; Vourakis et al., 2021) to biosynthesis-related (i.e., *Bacteroides*, and *Turicibacter*) bacteria (Granado-Serrano et al., 2019; Bourgin et al., 2020; Vourakis et al., 2021; Le et al., 2022) with a focus on TCHO-related metabolism, which was determined from the KEGG, was increased only after a high irradiance of chronic blue LED lighting in mice (*p*=0.007, **Figure 3B-2**). The ratio of light phase-induced bacteria (i.e., *Dehalobacterium*) (Li et al., 2021) to dark phase-induced bacteria (i.e., *Dorea* and *Anaerotruncus* spp.) (Li et al., 2021), however, was not significantly different at both a low and high irradiance of chronic blue LED lighting, but was decreased at a high irradiance of chronic white LED lighting (**Figure 3C-2**). Decreased beneficial bacteria and/or increased harmful or pathogenic bacteria can result in diseased conditions. Since it is hard to dissect six intersected genera into TCHO metabolism or CR regulation, the ratio of beneficial (i.e., *Lactobacillus, Coprococcus and Anaerostipes* spp.) (Lordan et al., 2020; Dempsey and Corr, 2022) to harmful (i.e., *Ruminococcus, Clostridium, and Anaeroplasma* spp.) (Shay et al., 2016) bacteria was determined. Intriguingly, there was decrease in the ratio of beneficial to harmful gut bacteria was decreased after a low irradiance of chronic blue LED lighting (*p*=0.001 and *q*=0.006, **Figure 3D-2**) but there was an increase in the ratio at a high irradiance (*p=*0.016 and *q*=0.034, **Figure 3D-1**). In the composition of TCHO metabolism, TCHO catabolism-related bacteria could be induced after a high irradiance of chronic blue LED lighting (*p*=0.020 and *q*=0.044, **Figure 3B-1**). Additionally, dark phase-induced bacteria were reduced after a high irradiance of chronic blue LED lighting (*p=*0.004 and *q*=0.025), **Figure 3C-1**). Importantly, harmful bacteria were found to be significantly increased at a low irradiance of blue LED lighting (*p*=0.040 and *q*=0.025), **Figure 3D-1**). Beneficial bacteria, however, were not changed at low irradiance of chronic blue LED lighting but were dramatically increased after a high irradiance of both blue and white LED lighting (*p*=0.011 and 0.002; *q*=0.026 and 0.034, **Figure 3D-1**). These results might indicate that a high irradiance of chronic blue LED lighting would increase beneficial bacteria to facilitate TCHO catabolism. The correlations between the obtained microbial biomarkers with blood TCHO in mice were further analyzed using Spearman’s correlation. Single bacteria, however, did not show any significant correlation with serum TCHO (**Supplementary Figure 3**). It is important to note that the ratio of beneficial to harmful gut bacteria was significantly and inversely correlated with serum TCHO, but not TG levels (*p*=0.041, **Figure 3E**). Moreover, the ratio of beneficial to harmful bacteria was positively correlated with that of TCHO catabolism to synthesis (*p*<0.001, **Figure 3E** Middle). The ratio of light to dark bacteria also showed a positive correlation with that of TCHO catabolism to synthesis (*p*=0.041, **Figure 3E** Right).

**Figure 4:**
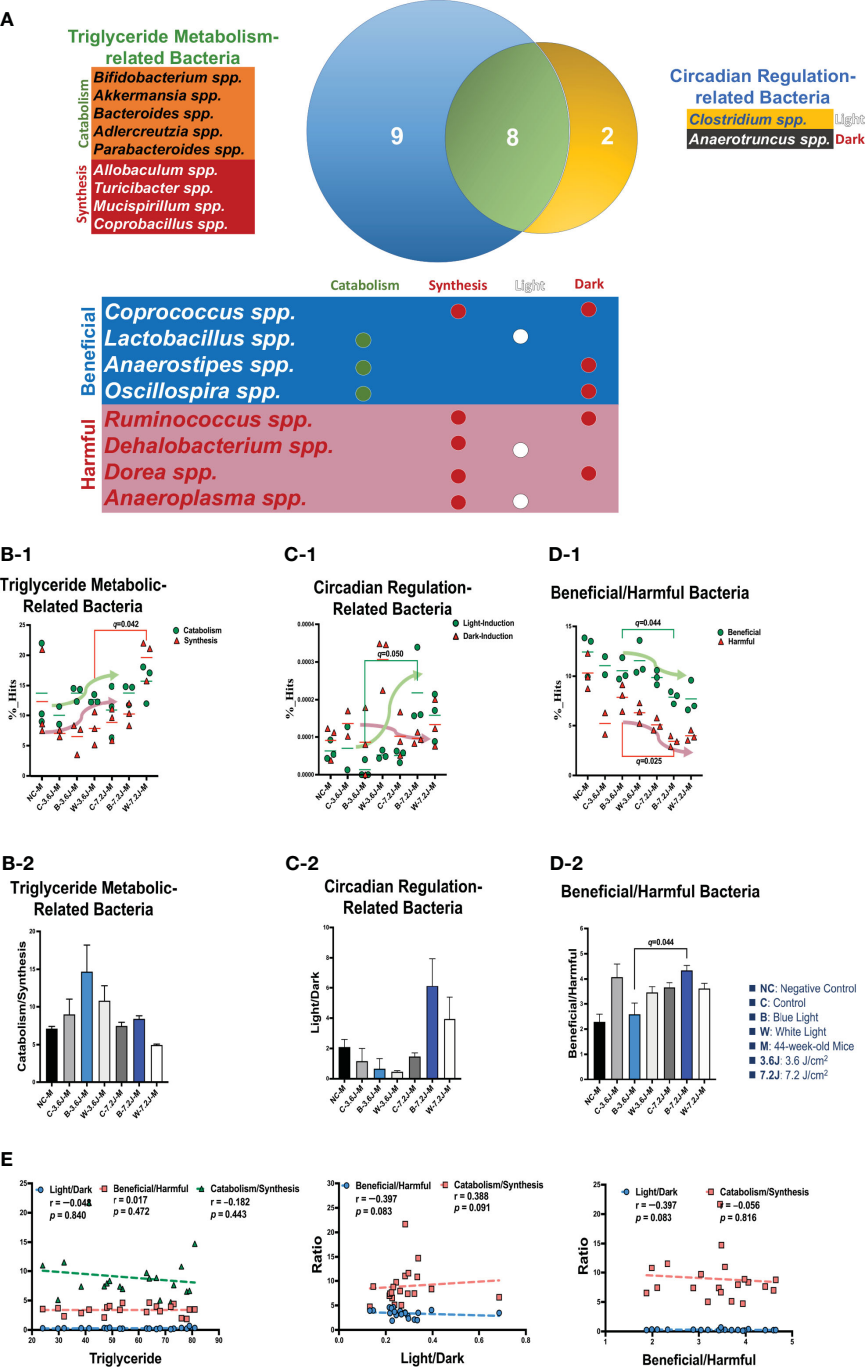
Chronic white LED lighting altered TG-metabolism bacteria. **(A)** A Venn plot of circadian regulation- and TG metabolism-related gut bacteria upon exposure to chronic LED lighting according to KEGG, level 2. There were nine bacteria in a blue circle presented as TG metabolism-related bacteria (in green color) (An et al., 2011), five bacteria belonged to TG catabolism (An et al., 2011; Wu et al., 2019), and four bacteria were related to TG synthesized (Yan et al., 2020; Zheng et al., 2021; Han et al., 2022). Two bacteria in a yellow circle gave as circadian regulation-related bacteria (in blue color) (Ruonan Yan and Zhang., 2022), and one bacterium was induced by light (Ruonan Yan and Zhang., 2022). The other one could be induced by dark (Voigt et al., 2014). Eight bacteria in a green circle were involved in both TG metabolism were related TG catabolism and five bacteria were in relation to synthesis and circadian regulation. Four bacteria were defined as beneficial bacteria (in blue color) (Tims et al., 2013; Goodrich et al., 2014), and the other four were defined as harmful bacteria (in red color) (Jin et al., 2019; Terzo et al., 2020). There were eight genera intersected in both circadian regulation and TG metabolism. Nine bacteria were independently involved in TG metabolism, including catabolism and synthesis. Two bacteria were independently influenced by circadian rhythms. **(B-1, B-2)** The bar plot showed the ratio of catabolism-to-synthesis of TG metabolism-related bacteria. The dot plot showed the changes of catabolism (green dot)/synthesis (red triangle) bacteria after LED lighting. **(C-1, C-2)** The bar plot showed the ratio of the light-induction to dark induction of circadian-regulated bacteria. The dot plot showed light-induced (green dot)/dark-induced (red triangle) bacteria after exposure to LED lighting. **(D-1, D-2)** The bar plot showed the ratio of beneficial to harmful bacteria which were both involved in circadian regulation and TG metabolism. The dot plot showed the change in beneficial (green dot)/harmful (red triangle) bacteria after exposure to LED lighting. The dot plot showed the altered abundance of beneficial (green dot)/dark-induced (red triangle) bacteria after chronic LED lighting. **(E)** The ratio of beneficial to harmful bacteria did not show any significant correlation with serum TG (left). Three was any correlation between three ratios (middle and right). All the results were correlated by Benjamini-Hochberg correlation methods. A *q-*value less than 0.05 was considered statistically significant.

Serum TG levels were also analyzed using the same strategy. The Venn diagram plot showed that two genera were independently involved in circadian regulation and nine genera (*Bifidobacterium, Akkermansia, Bacteroides, Adlercreutzia, Parabacteroides, Allobaculum, Turicibacter, Mucispirillum* and *Corobacillus* spp. in the blue circle, **Figure 4A**) were independently included in TG metabolism (**Figure 4A**). There were eight genera (*Coprococcus, Lactobacillus, Anaerostipes, Oscillospira, Ruminococcus, Dehalobacterium, Dorea* and *Anaeroplasma* spp. in the green circle, **Figure 4A**) intersected in both circadian regulation- and TG metabolism-related gut bacteria (**Figure 4A**). Chronic white LED lighting significantly decreased the ratio of the catabolism (i.e., *Bifidobacterium, Akkermansia, Bacteroides, Adlercreutzia* and *Parabacteroides* spp.) (An et al., 2011; Wu et al., 2019) to the synthesis (i.e., *Allobaculum, Turicibacter, Mucispirillum* and *Corprobacillus* spp.) (Yan et al., 2020; Zheng et al., 2021; Han et al., 2022) of TG at high irradiance (*p*=0.007, **Figure 4B-1**). Both bacteria related to the catabolism and synthesis of TG were increased at high irradiance (**Figure 4B-2**). In circadian regulation-related bacteria, the ratio of light (i.e., *Clostridium* spp.) (Ruonan Yan and Zhang., 2022) to dark (i.e., *Anaerotruncus* spp.) (Voigt et al., 2014)-induced bacteria was dramatically increased at a high irradiance of both blue and white LED lighting but did not reach statistical significance (**Figure 4C-2**). Light-induced bacteria were slightly increased at a high irradiance of white LED lighting but did not reach statistically significant (**Figure 4C-2**). There was no significant difference in the ratio of beneficial to harmful bacteria upon exposure to chronic LED lighting regardless of low and high irradiance (**Figure 4D**). Both beneficial (i.e., *Coprococcus, Lactobacillus, Anaerostipes*, and *Oscillospira* spp.) (Tims et al., 2013; Goodrich et al., 2014) and harmful (i.e., *Ruminococcus, Dehalobacterium, Dorea*, and *Anaeeroplasma* spp.) (Jin et al., 2019; Terzo et al., 2020) bacteria intersected between circadian regulation and TG metabolism were significantly decreased at a high irradiance of blue LED lighting (*p*=0.024 and 0.025, respectively; **Figure 4E** Left). Nevertheless, the ratio of beneficial to harmful bacteria did not show any correlation with that of TG catabolism to synthesis (*p*=0.091, **Figure 4E** middle). Meanwhile, the ratio of light to dark did not show any correlation with that of TG catabolism to synthesis (*p*=0.816, **Figure 4E** right). In short, a high irradiance of chronic blue LED lighting triggered a dramatic increase in the ratio of beneficial to harmful gut bacteria at high irradiance, which might antagonize a heavier optic stress-related CR disruption.

### Chronic blue LED lighting changed the ratio of beneficial to harmful bacteria which was positively correlated with bile acid biosynthesis

**Figure 5:**
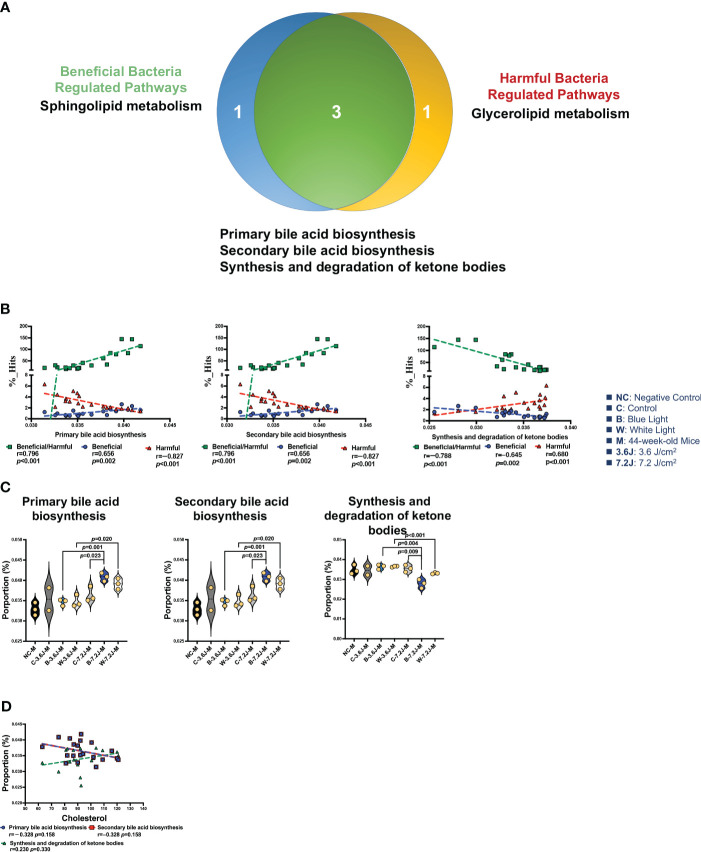
Chronic LED lighting-modulated changes of the gut microbiota composition were related to bile acid biosynthesis. **(A)** A Venn plot of beneficial and harmful bacteria-regulated metabolism pathways. Primary and secondary bile acid biosynthesis as well as the synthesis and degradation of ketone bodies were regulated with beneficial and harmful bacteria. **(B)** The ratio of beneficial/harmful bacteria was positively correlated with primary and secondary bile acid biosynthesis but was negatively correlated with the synthesis and degradation of ketone bodies. **(C)** The abundances of three pathways regulated by both beneficial and harmful bacteria were represented. **(D)** Primary and secondary bile acid biosynthesis pathways showed a negative correlation margin with the serum TCHO level. A *p* value less than 0.05 was considered statistically significant.

There were 12 and 13 function pathways associated with beneficial and harmful bacteria, respectively (**Supplementary Figure 4**). Three pathways were regulated by both beneficial and harmful bacteria (**Figure 5A**). Primary and secondary bile acid biosynthesis showed a positive correlation with the ratio of beneficial to harmful bacteria (r=0.796, *p*<0.001, **Figure 5B**). The synthesis and degradation of ketone bodies, however, were negatively correlated with the ratio of beneficial to harmful bacteria (r=−0.788, *p*<0.001, **Figure 5B**). Bile acid biosynthesis pathways (both primary and secondary bile acid biosynthesis) were significantly increased after a high irradiance of blue LED lighting compared to the control (both *p*=0.023, **Figure 5C**). It is noted that bile acids were synthesized from TCHO in the liver (Russell, 2003). Increased bile acid biosynthesis might be the key regulator in serum TCHO after a high irradiance of blue LED lighting. Although there was broader significance, these two pathways showed an inverse correlation with serum TCHO (r=-0.328, p=0.158, and 248 r=0.230, p=0.330, respectively; **Figure 5D**), suggesting that beneficial bacteria might activate primary and secondary bile acid biosynthesis and in turn down-regulate serum TCHO levels in mice. In summary, a low irradiance of chronic blue LED lighting could increase serum TCHO levels and also promoted an increase in harmful bacteria. A high irradiance of chronic blue LED lighting would enrich beneficial bacteria to facilitate TCHO catabolism through inducing primary and secondary bile acid biosynthesis. These phenomena would protect the host from the risk of cardiovascular disease in relation to the elevation of serum TCHO levels.

## Discussion

Chronic exposure to LED lighting might lead to circadian disruption, recruit gut dysbiosis and subsequently harvest disease niches. The term ‘chronic’ is applied when the time course of the disease lasts for more than three months in humans (Bernell and Howard, 2016). We found that chronic, persistent exposure with a high irradiance of blue LED light significantly decreased α-diversity, but not β-diversity, after 33 weeks. Thirty-three weeks in adult mice is about 18.84 years in the life of a human adult (Wang et al., 2020). A lower α-diversity of the gut microbiota has been associated with such disorders and diseases as obesity, diabetes, colorectal cancer and children suffering from epilepsy (Mosca et al., 2016; Kriss et al., 2018; Zhang et al., 2018). Importantly noted, a high irradiance of blue LED light also significantly decreased the α-diversity at 27 weeks. Chronic exposure to blue LED lighting might harvest the niche of metabolic disorders and also increase an inflammatory gut environment early, at least in mice.

Although most studies have proposed that a short period of light-mediated circadian dysregulation is linked to glucose dysregulation and insulin resistance (Thaiss et al., 2014; Grosbellet et al., 2015; Figueiro et al., 2017), there is still an absence of information regarding the impacts of a long-lasting (up to a decade) of exposure to LED lighting on human health. The frequency of night shifts appears to affect short-term physiological effects and is suggested to also affect long-term health (Garde et al., 2020). A higher number of night shifts per month has been found to be positively associated with obesity (Peplonska et al., 2015; Ramin et al., 2015; Qiao et al., 2020). However, people working longer than 20 years in night shift work appear to have a lower level of LDL and showed a decrease in the rates of overweightness and obesity (Pan et al., 2011; Loef et al., 2019). Our results revealed that blue light would harvest gut dysbiosis and specifically result in TCHO dysregulation. Consistently, recent studies have indicated night shift workers have increased TCHO levels, decreased HDL levels and altered gut microbiota composition in humans (Akbari et al., 2015; Dutheil et al., 2020; Mortas et al., 2020). In addition, we found that most of the bacteria (16/19) altered by chronic LED lighting were related to lipid metabolism. Accordingly, chronic LED lighting specifically alters serum lipid but not glucose levels in mice. Moreover, a low irradiance of both chronic blue and white LED lighting significantly increased TCHO but not TG levels. It is important to note that white LED light consists of 19~21% of blue light (Dieleman et al., 2019). In this experiment, white LED containing 17.4% of blue light was used. Hence, when the microbial abundance was altered by both white and blue LED light, it is rational that the impacts of blue LED light are dominant on the abundance of specific microbiomes if significant result were present in blue LED light-mediated alterations.

Our results revealed that chronic LED lighting would significantly change the abundances of 8 gut bacteria by specific wavelengths among 19 gut microbes. Although these critical bacteria might reflect the aging process or were modulated by experimental factors such as cage shifts, the change in the core bacteria was not the result of aging and cage shifts because the trend upon LED lighting was not in alignment with the trends related to aging. For example, *Ruminococcus* spp. were increased along with increasing weeks/age, but reduced at a high irradiance of blue LED lighting (**Supplementary Figure 2**). Therefore, blue LED lighting might in major contribute to the reduced abundance of *Ruminococcus* spp., which are also less abundant in people with inflammatory bowel disease (IBD) (Nagao-Kitamoto and Kamada, 2017) and in patients with Parkinson’s disease (Hill-Burns et al., 2017). Hence, chronic blue LED lighting may contribute to inflammatory niches in the gut. Intriguingly, *Lactobacillus siliginis* were dramatically decreased at a low irradiance of blue LED lighting but were elevated after high irradiance (**Supplementary Figure 5**). *Lactobacillus hayakitensis*, however, showed a significant increase at a high irradiance of both blue and white LED lighting (**Supplementary Figure 5**). These results imply that *Lactobacillus* spp. might be beneficial to hypercholesteremia induced by LED lighting-related CR dysregulation. Additionally, noted, *Lactobacillus* are the only beneficial bacteria responsible for the catabolism of both lipids, TCHO and TG, and can be induced by light at the same time. Chronic blue LED lighting might enrich their abundance to tune CR disruption. It has been suggested that blue-enriched light can be used by nightshift workers to optimize their body rhythm (Motamedzadeh et al., 2017; Sletten et al., 2017; Sletten et al., 2021).

Our results showed that a low irradiance of both blue and white LED light could increase serum TCHO. There were six genera intersected in both circadian regulation- and TCHO metabolism-related gut bacteria. Single bacteria did not show any significant correlation with serum TCHO (**Supplementary Figure 3**). It was found that the ratio of beneficial to harmful bacteria in six bacteria was decreased after a low irradiance of blue LED lighting, whereas this ratio was significantly increased at high irradiance of blue LED lighting. A high irradiance of chronic blue LED lighting could increase beneficial bacteria, leading to an increased ratio of beneficial to harmful bacteria. The increased beneficial bacteria including *Coprococcus*, *Anaerostipes*, and *Lactobacillus* spp. have been demonstrated in lowering TCHO (Ren et al., 2019; Tarrah et al., 2021; Vourakis et al., 2021) and therefore might facilitate TCHO catabolism. A low irradiance of blue LED light could increase harmful bacteria but did not affect beneficial ones, resulting in a decreased ratio of beneficial to harmful bacteria; *Clostridium* and *Ruminococcus* spp., have been involved in increasing TCHO (Graber and Boltjes, 1968; Falony et al., 2015; Bo et al., 2017). In human, *Ruminiococcus* spp. is also elevated in the night shift workers (Mortas et al., 2022). It is noted the irradiance of blue light in modern electronic devices is 0.008 to 0.230 Watt/m^2^ (Moyano et al., 2020). In our study, the irradiance of each light is 3.6 J/cm^2^ and 7.2 J/cm^2^ (1 Watt = 1 Joule/sec). The energy of low irradiance is higher by about 125 times than modern electronic devices do. Hence, in the real world, a long-lasting exposure to general blue LED lighting (i.e., near to a low irradiance in our study) at night may play a predisposing role in harvesting hypercholesterolemia related disorders in humans. Thus, in the real world, a chronic exposure to blue light might accumulate the energy nearing low irradiance in our study. Our study illustrated one of the impacts of chronic exposure to blue LED light might be relatively limited on TCHO regulation via harvesting gut dysbiosis in night shift workers if they don’t have other confounding factors such as a high-fat diet. Intriguingly noted, the ratio used in our study means a dynamic balance between two sets of bacteria with known opposite effects toward one target as seen in the case of *Firmicutes/Bacteroidetes* (F/B) ratio and *Prevotella/Bacteroides* (P/B) ratio (Gorvitovskaia et al., 2016; Stojanov et al., 2020). These bacteria included in our study might participate in both circadian regulation and TCHO metabolism but have either benefits or harms to human or mice health. The ratio of light- to dark-induced bacteria was also found negatively correlated with total TCHO, but not TG, as seen in that of beneficial to harmful bacteria. Taken together, a long-lasting exposure to low-irradiance of blue LED lighting may be one of the predisposing factors for hypercholesterolemia in human.

Our results strengthen the consensus that there is a complex inter-bacteria network, and not only a single bacterium, in the modulation of metabolism homeostasis between microorganisms and the host; this can be seen in the *Firmicutes* to *Bacteroidetes* (F/B) ratio, which contributes to a dozens of diseases, such as obesity, and inflammatory bowel diseases (Stojanov et al., 2020). More intriguingly, both primary and secondary bile acid biosynthesis were positively correlated with the ratio of beneficial to harmful bacteria, and were significantly increased after a high irradiance of blue LED lighting. There were borderline negatively significant correlations between bile acid synthesis and serum TCHO. Taken together, a low irradiance of chronic blue LED lighting promoted an increase in harmful bacteria leading to a lower beneficial to harmful bacteria ratio which resulted in the upregulation of serum TCHO levels through reduced bile acid synthesis. Furthermore, a high irradiance of chronic blue LED lighting enriched beneficial bacteria to facilitate TCHO catabolism through increased bile acid biosynthesis. As bacterial enzymes that conjugate bile acids vary according to bacterial strain, the microbial community is a key determinant of bile acid pool size and composition (Mazuy et al., 2015; Staley et al., 2017). Bile acids themselves also affect the composition of the gut microbiome, via direct detergent effects on bacteria as well as effects on intestinal mucosa integrity (Trauner et al., 2013). A previous study showed that the antioxidant tempol preferentially reduced the relative abundance of the *Lactobacillus* genus, decreased bile salt hydrolase (BSH) activity, and led to changes in bile acids composition that ultimately improved obesity (Sayin et al., 2013).

The major aim of our study is to explore the impacts of chronic exposure to blue LED lighting, a well-known disruptor of circadian rhythm and a light source of modern electric devices, on gut microbiota. In view of wavelength, a higher wavelength of light like red instead of white which itself contains blue could be an appropriate control. However, as for the impacts of circadian rhythm, white light is a better control for blue light in the acritical light at night (ALAN) model, mimicking night shift workers. Red light has rare effects on the circadian clock in human (Tahkamo et al., 2019) and mice (Zhang et al., 2017). Also, the effects of white and red light did not show any significant difference of circadian disruption in a goose model (Li et al., 2020). Although the n number is low in the present study, the Weighted UniFrac distance and distribution patterns of 3 samples from the same group of each treatment were quite similar. Besides, we have also utilized the Benjamini-Hochberg false discovery rate (FDR) method to correlate our results and minimalized the error. Our novel findings included: (1) a high irradiance of blue LED lighting decreased alpha diversity; (2) at low irradiance, chronic blue LED lighting increased the abundance of harmful bacteria, leading to a decreased ratio of beneficial to harmful bacteria, and might subsequently increase serum TCHO levels; (3) at high irradiance, chronic blue LED lighting increased beneficial bacteria as well as the ratio of beneficial to harmful bacteria which in turn reduced serum TCHO; and (4) the ratio of beneficial to harmful bacteria was negatively correlated with serum TCHO but was positively correlated with bile acid biosynthesis. Our results showed for the first time that chronic blue LED lighting could harvest gut dysbiosis and specifically dysregulate TCHO metabolism in mice.

The present study shed the first insight into the impacts of long-term exposure to blue and white light on gut microbiota without any confounding factor in mice. In the real world, long-term night-shift workers frequently have increased blood TCHO and dyslipidemia (Dutheil et al., 2020). Night shift workers, who sleep less than 6 hours and work for at least 15 days also have a three times obesity rate compared to day-shift workers independent of age and gender (Brum et al., 2020). Although the cases are limited, night work indeed changes gut microbiota consistent with elevated risk for future metabolic and gastrointestinal pathology (Mortas et al., 2020). Our observations require further investigations in a larger cohort of night shift workers. Herein, we recommend that night shift workers should be avoided of blue LED lighting from blue light-based electric devices for a long and lasting time.

## Materials and methods

### Animals

Wild-type male C57BL/6 mice were purchased from the National Laboratory Animal Center, National Applied Research Laboratories, Taiwan. Mice were housed in a temperature (22-26 °C)- and humidity (40-60%)-controlled room with a 12 hours light/12 hours dark cycle (Banks et al., 2022). Food and water were supplied ad libitum accessing to normal chow. All animal protocols were approved by the Institutional Animal Care and Use Committee of the National Health Research Institutes accredited by the AAALAC International (Protocol No: NHRI-IACUC-104125AE). Previous studies have showed that circadian of mice could be easily regulated (Opperhuizen et al., 2015; Capri et al., 2019; Banks et al., 2022). C57BL/6 mice being with normal melanin and hair were the suitable animal model for circadian rhythms study. The use of mice to study shift-work allows a control of the environmental factors including the genetic background, and also focuses on the direct effect of the shift-work pattern.

### Light irradiation

Two customized light boxes with light-emitting diodes (LED) of adjustable light intensity on the ceiling were used. LEDs of blue light (473 ± 12.0 nm), and white light (containing around 17.4% blue light) were used in this study. 9-week-old mice were randomly assigned to 4 groups (NC: in the origin cage; C: move to the light box without light; W-3.6J/7.2J: 3.6 J/cm^2^ or 7.2 J/cm^2^ of white light irradiation for 1hr/2hrs; B-3.6J/7.2J: 3.6 J/cm^2^ or 7.2 J/cm^2^ of blue light irradiation for 1hr/2hrs) and allowed to adopt the environment for 2 weeks. Mice were exposed five days a week to the blue light irradiation (3.6 J/cm^2^ or 7.2 J/cm^2^, each N=3, total N=6) or to the white light irradiation (3.6 J/cm^2^ or 7.2 J/cm^2^, each N=3, total N=6) at ZT13.5-14 being equal to AM 9:30-10:00 under un-anesthetized condition. Mice given the same treatments without the light on was defined as the control group (C, N=3) and in the origin cage without movement was defined as the negative control group (NC, N=3). The average lifespan of experimental mice is about 24 months (Wilkinson et al., 2012), whereas the life of humans globally is about 80 years. According to this observation, the average one human year is equal to 9.125 mice days (Dutta and Sengupta, 2016). Based on this formula, 11-week-old mice are equal to humans at 8.4 years old; 27-week-olds are equal to 20.7 years old, and 44-week-olds are equal to 33.8 years old.

### 16s v3v4 rDNA amplicon sequencing

Fecal samples were collected at ZT18 being equal to PM 2:00 and all specimens were extracted using Qiagen DNA kit according to the manufacturer’s instructions. DNA samples with OD 260/280 in the range of 1.8~2.0 for further 16s rDNA amplicon PCR. 16S rDNA PCR was using metagenomic DNA as a template was amplified with the bacterial-specific primers S17 (5’-TCG TCG GCA GCG TCA GAT GTG TAT AAG AGA CAG CCT ACG GGN GGC WGC AG-3’) and A21 (5’-GTC TCG TGG GCT CGG AGA TGT GTA TAA GAG ACA GGA CTA CHV GGG TAT CTA ATC C-3’). Fragment Analyzer M5330AA (Agilent Technologies) checked the amplified DNA sizing. Sequencing was carried out by the Illumina MiSeq platform. DNA samples were attached to indices and Illumina sequencing adapters using the Nextera XT Index Kit. After library construction, samples were mixed with MiSeq Reagent Kit v3 (600-cycle) and loaded onto a MiSeq cartridge, then onto the instrument. Automated cluster generation and a 2x300 bp paired-end sequencing run were performed. The sequences generated went through a filtering process to obtain the qualified reads. Total reads were merged, remove low-quality sequences, remove chimera sequence and cluster OTU at 97% similarity with the Greengenes database (v13.8). All OTU sequences and diversity analysis were using CLC Microbial Genomics Module (v10.0, Qiagen, Germany), BaseSpace (Illumina, USA), and Graphpad Prism 9 (v9.1.1 Graphpad Software, USA).

### Biochemistry analysis

Blood samples were collected via submandibular blood collection after 44 weeks of lighting. Blood samples were centrifuged at 3,000 rpm for 30 mins and harvested serum for blood TCHO, TG, and glucose analysis. According to the manufacturer’s instructions, blood TCHO, TG, and glucose were analyzed using Fuji NX500. Data were expressed as mean ± standard error of the mean. Generalized model analysis was used to evaluate the relationships between light exposure, white or blue light, TCHO, TG, and glucose. Statistical significance was set at *p* < 0.05. Statistical analysis was performed using the GraphPad Prism software (v9.1.1 GraphPad Software, USA). The %_Hits was the percentage of specific bacteria after normalized to total reads in each specimen.

### Bioinformatics analysis

Bioinformatics was performed using the CLC Microbial Genomics Module. Alpha diversity was measured using Shannon index, which calculates the overall diversity of each group including the number of observed species (Richness) and how evenly of observed taxonomic (Evenness). Beta diversity was measured using PCoA-Weighted UniFrac, which determines the difference of microbial composition between groups. Hierarchical clustering of the top 25 abundance of OTU taxonomic were deduced using a heatmap to determine distribution patterns between groups. OTU table was generated by CLC Microbial Genomics Module and further analyzed with Linear discriminant analysis Effect Size (LEfSe) and Phylogenetic Investigation of Communities by Reconstruction of Unobserved States (PICRUSt) analysis. LEfSe was performed by the website of Galaxy/HutLab to identify specific microbial markers between groups with an alpha value for the factorial Kruskal-Wallis test/pairwise Wilcoxon test of 0.05 and LDA score cut-off of 2.0. PICRUSt prediction was performed by the website of Galaxy according to the Kyoto Encyclopedia of Genes and Genomes (KEGG) functional pathways database and analyzed with Statistical Analysis of Metagenomic Profiles (STAMP, v2.1.3) software. The criteria of STAMP were set up with remove unclassified reads, *p*<0.01, and effect size 0.2. The results revealed those functional pathways with a significantly different abundance at level 3 between groups. Spearman’s correlation and principal component analysis (PCA) were utilized by R language (v4.0.2). Comparison of different groups was performed by t-test with two-tailed. A p-value less than 0.05 were considered statistically significant.

### Statistics analysis

Statistical analysis was also performed using Microsoft Excel software and GraphPad Prism software (v9.1.1 Graphpad Software, USA). Graphs and tables represent the mean ± standard error of the mean (SEM) obtained from two or three independent experiments and collected from 3 animals in each group. Differences were evaluated using one-way ANOVA. Multiple comparisons were performed by Duncan’s new multiple range test (MRT). Results with *p*-values of less than 0.05 were considered significant. The Benjamini–Hochberg false discovery rate (FDR) correction for multiple comparisons at each level was applied separately. FDR (*q*-value) <0.05 was considered significant.

## Data availability statement

The datasets presented in this study can be found in online repositories. The names of the repository/repositories and accession number(s) can be found in the article/**Supplementary Material**.

## Ethics statement

The animal studies were approved by Institutional Animal Care and Use Committee of the National Health Research Institutes accredited by the AAALAC International. The studies were conducted in accordance with the local legislation and institutional requirements. Written informed consent was obtained from the owners for the participation of their animals in this study.

## Author contributions

C-HH: Formal Analysis, Visualization, Writing – original draft. SY: Conceptualization, Methodology, Writing – review & editing. H-ShY: Data curation, Methodology, Writing – review & editing. H-PT: Investigation, Methodology, Writing – review & editing. Y-TY: Project administration, Supervision, Writing – review & editing. H-SuY: Conceptualization, Funding acquisition, Project administration, Validation, Writing – review & editing.
