# Supplementary material for: Chronic blue light-emitting diode exposure harvests gut dysbiosis related to cholesterol dysregulation
Source: Front Cell Infect Microbiol. 2024 Jan 8;13:1320713. doi: 10.3389/fcimb.2023.1320713 (PMC10800827; doi:10.3389/fcimb.2023.1320713)
Supplement: Supplementary file 1 [file DataSheet_1.docx]

**Supplemental Material**

**A**


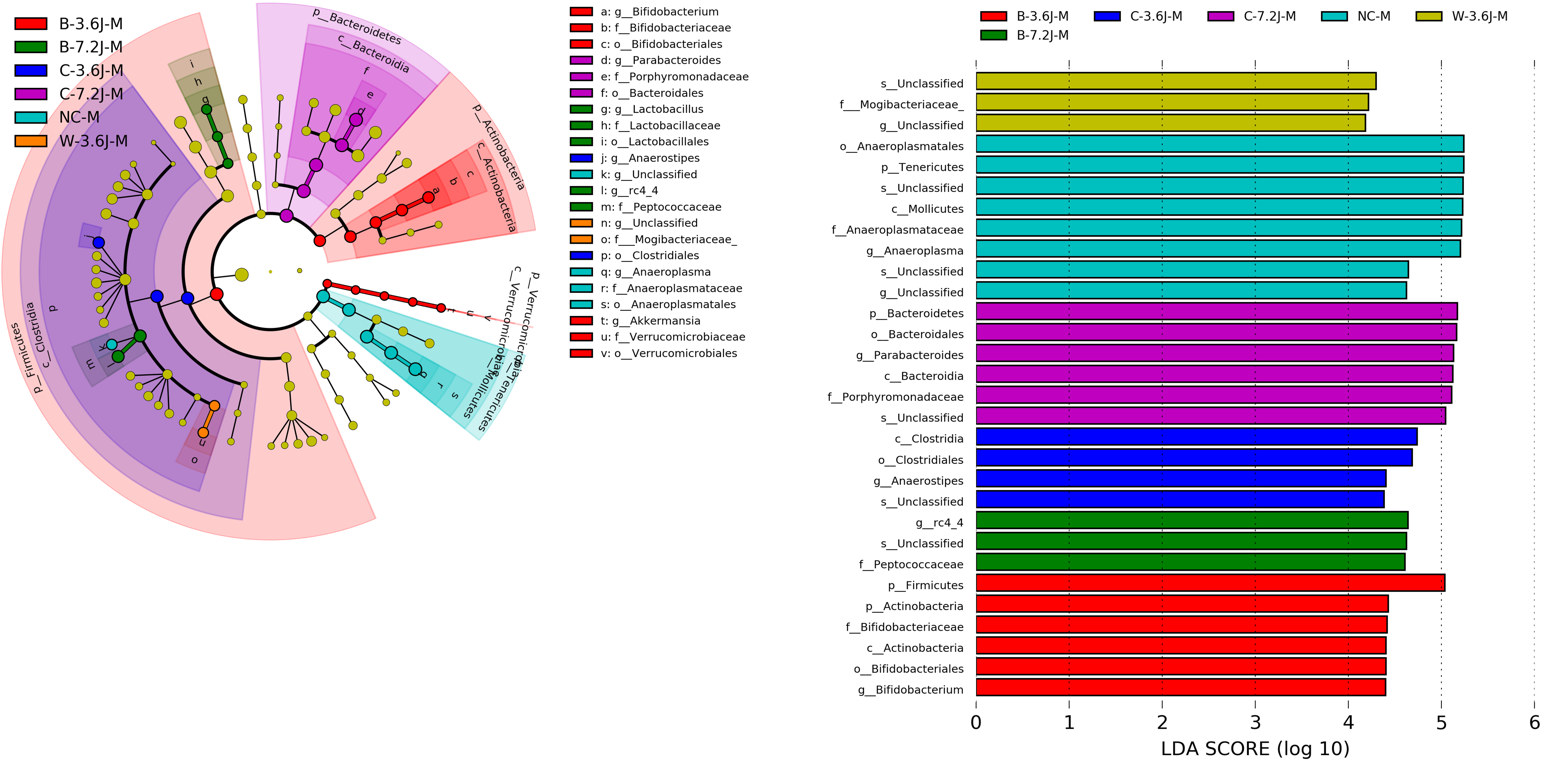


**B**


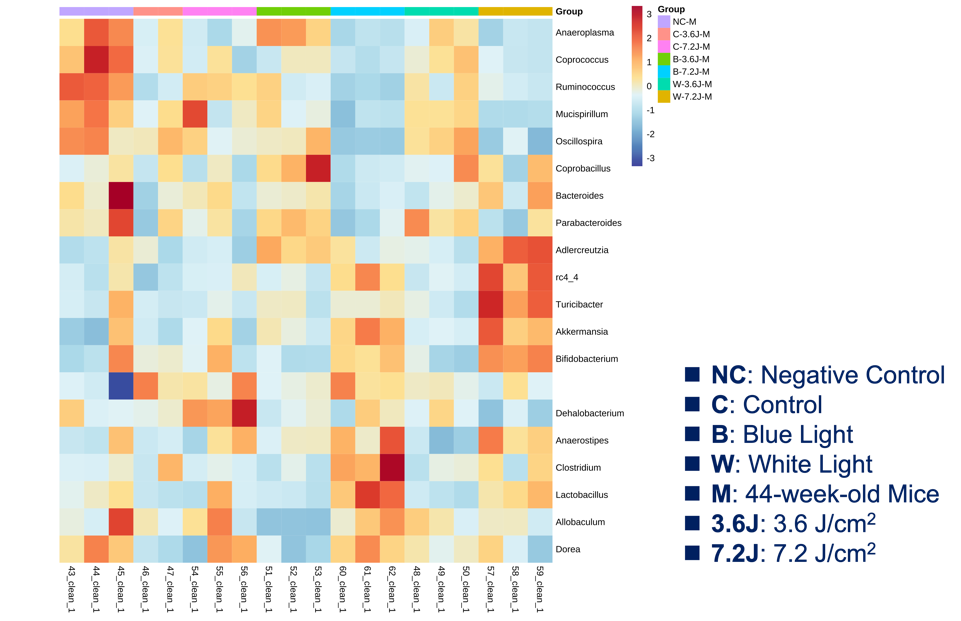


**Figure S1. Core bacteria changed after chronic blue and white LED lighting in mice.** (A) The core microbiota analysis was determined by LEfSe (linear discriminant analysis (LDA) effect size) after 44 weeks. A cladogram plotted from the LEfSe analysis showed the taxonomy levels. In the graph, the object from the center of the circumference shows kingdom, phylum, class, order, family, and genus, respectively. The bar plot showed the enriched microbiome associated with different irradiances and wavelengths of LED. The threshold on the logarithmic LDA score for discriminative features was set to 2.0. (**B)** The core bacteria analysis was determined by a heatmap. A heatmap diagram of the gut microbiota composition was shown at the genus level for all levels of irradiance of LED lighting.


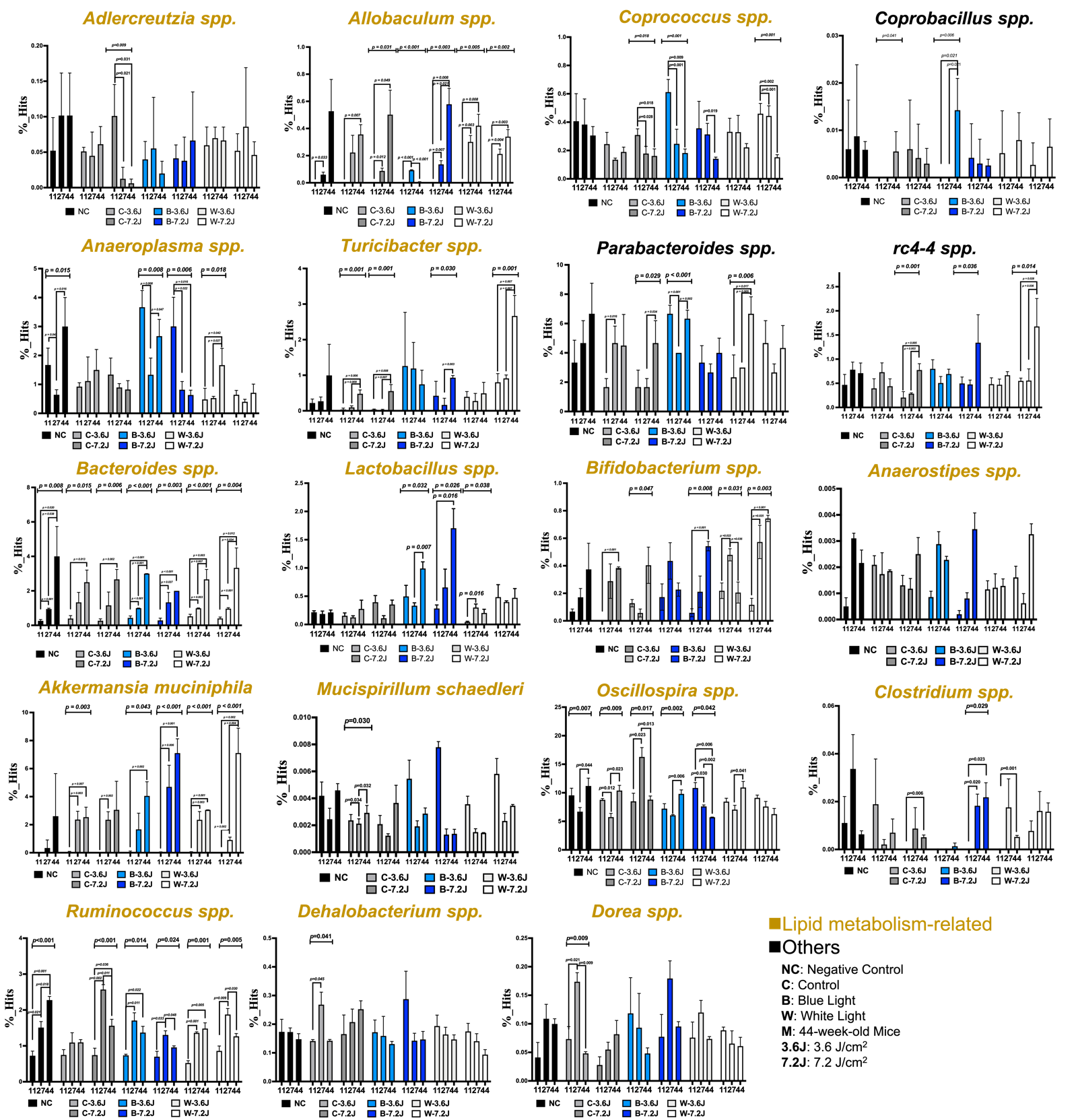


**Figure S2. Core bacteria changed upon the low and high irradiance of white and blue lighting across different time points.** The bar plot showed the relative abundance of lipid metabolism-related bacteria upon two irradiances of LED lighting from 11 weeks to 44 weeks. One-way ANOVA was used to compare groups 11 weeks before LED lighting. A *p* value of less than 0.05 was considered statistically significant.


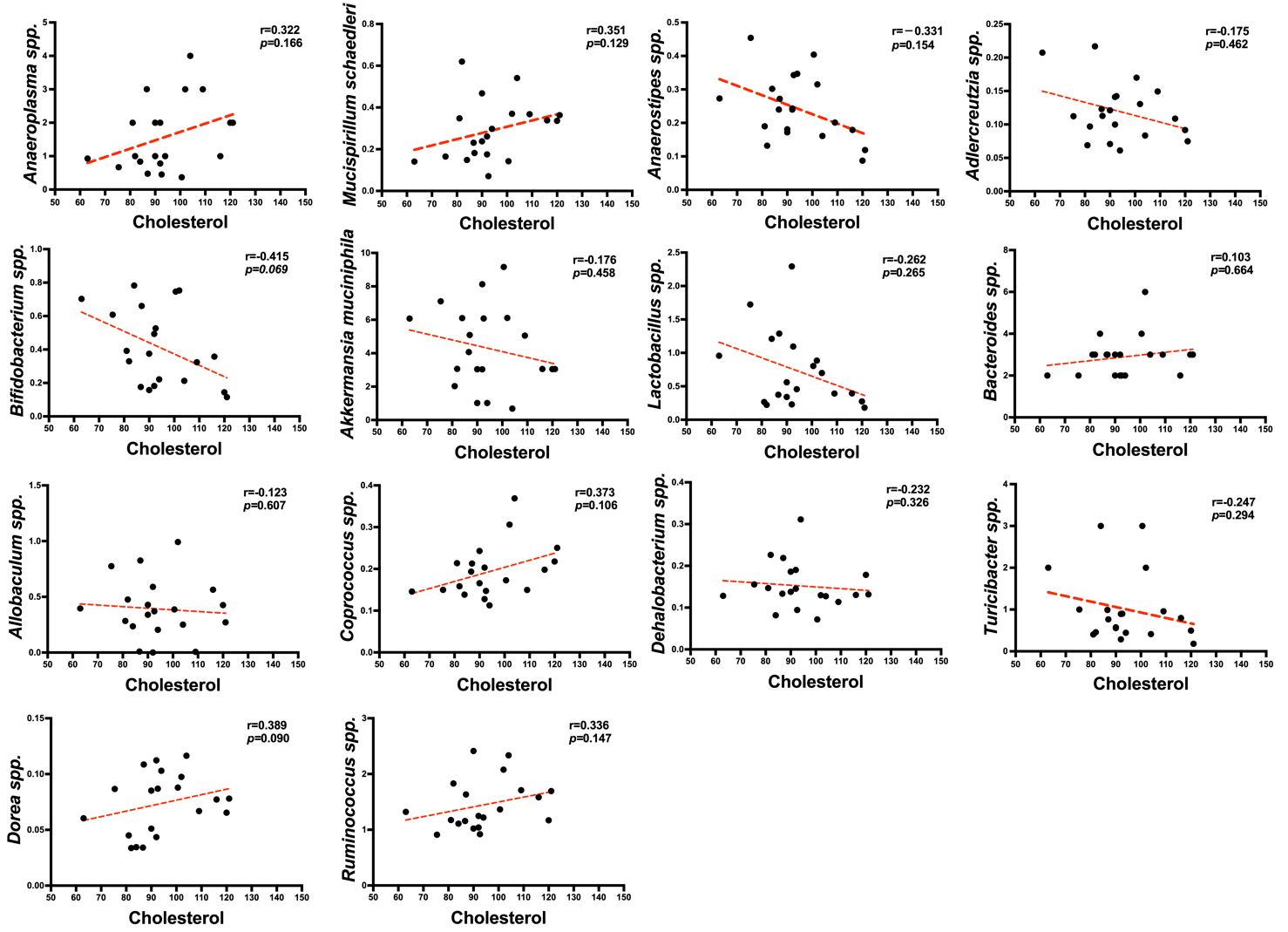


**Figure S3. Correlation between the core bacteria and serum cholesterol level.** The dot plot showed the correlation of each bacterium which participated in lipid metabolism with the serum cholesterol level. All lipid metabolism-related bacteria did not show any significant correlation with the serum cholesterol. A *p* value less than 0.05 was considered statistically significant.

**A**


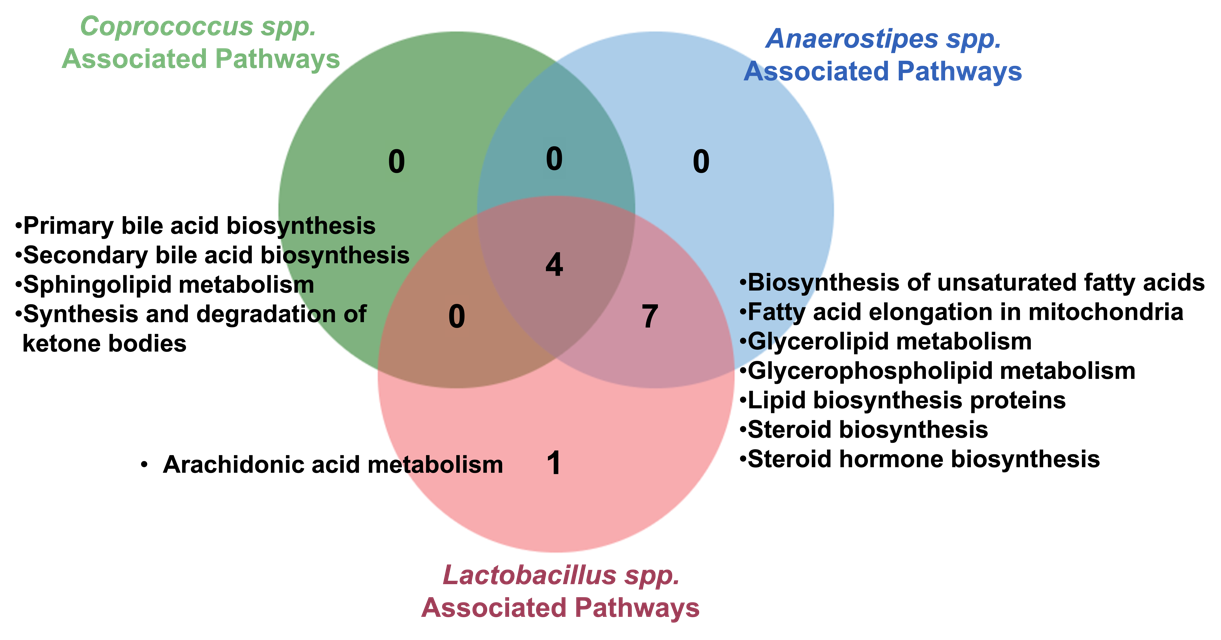


**B**


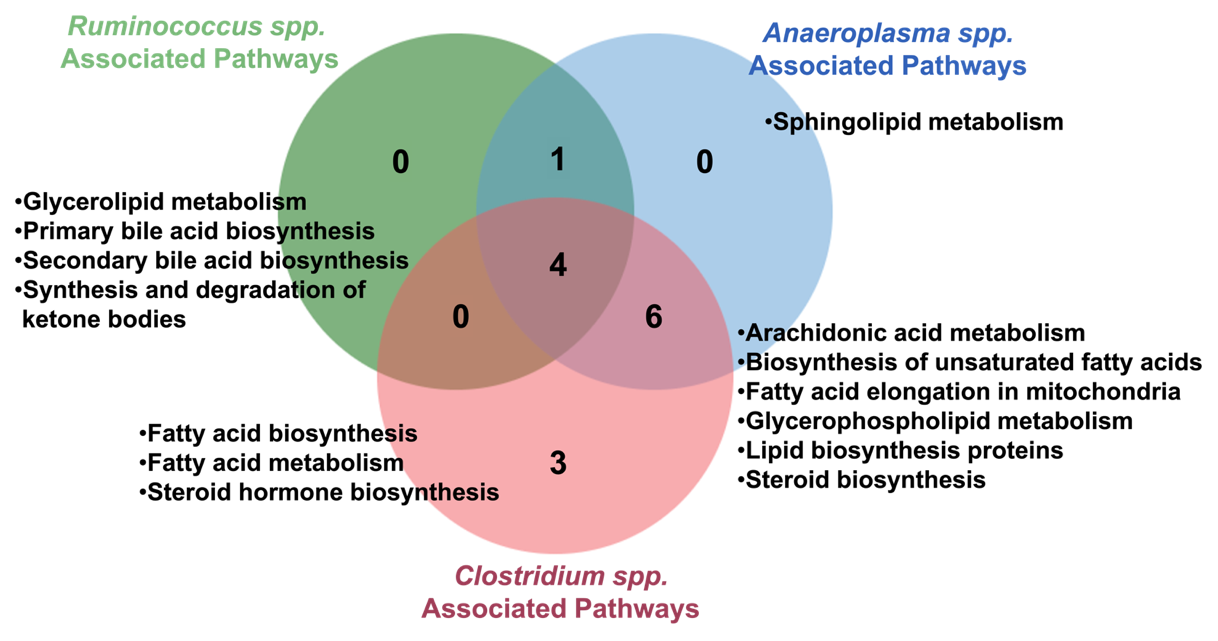


**Figure S4. The signaling pathways regulated by circadian regulation- and cholesterol metabolism-related bacteria.** (A) The Venn plot showed the signaling pathways regulated by the beneficial bacteria of the cholesterol metabolism. Four signaling pathways, including primary and secondary bile acid biosynthesis, the synthesis and degradation of ketone bodies, and sphingolipid metabolism, were regulated by beneficial bacteria. (B) The Venn plot showed the signaling pathways regulated by harmful bacteria. Four signaling pathways, primary and secondary bile acid biosynthesis, the synthesis and degradation of ketone bodies, and glycerolipid metabolism, were regulated by harmful bacteria.

**A**


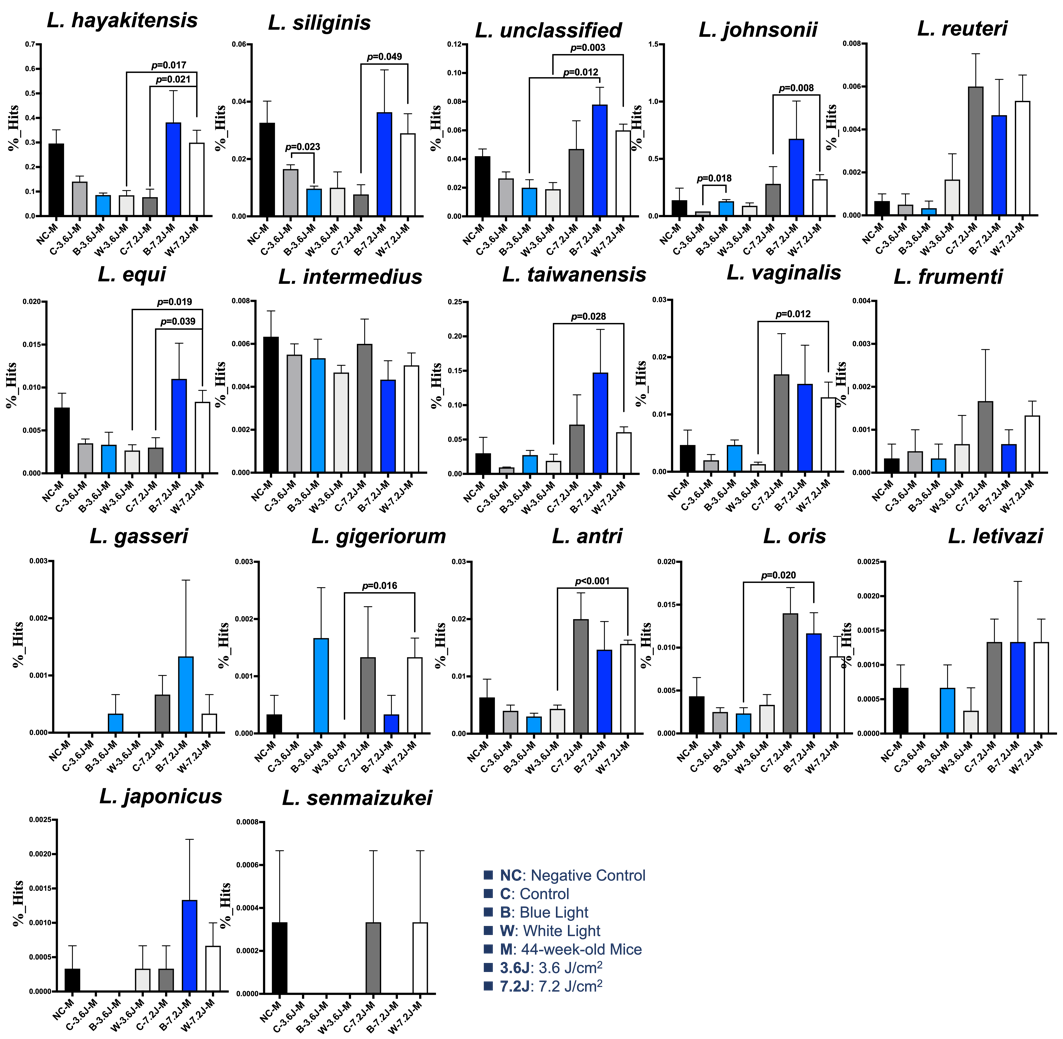


**B**

**
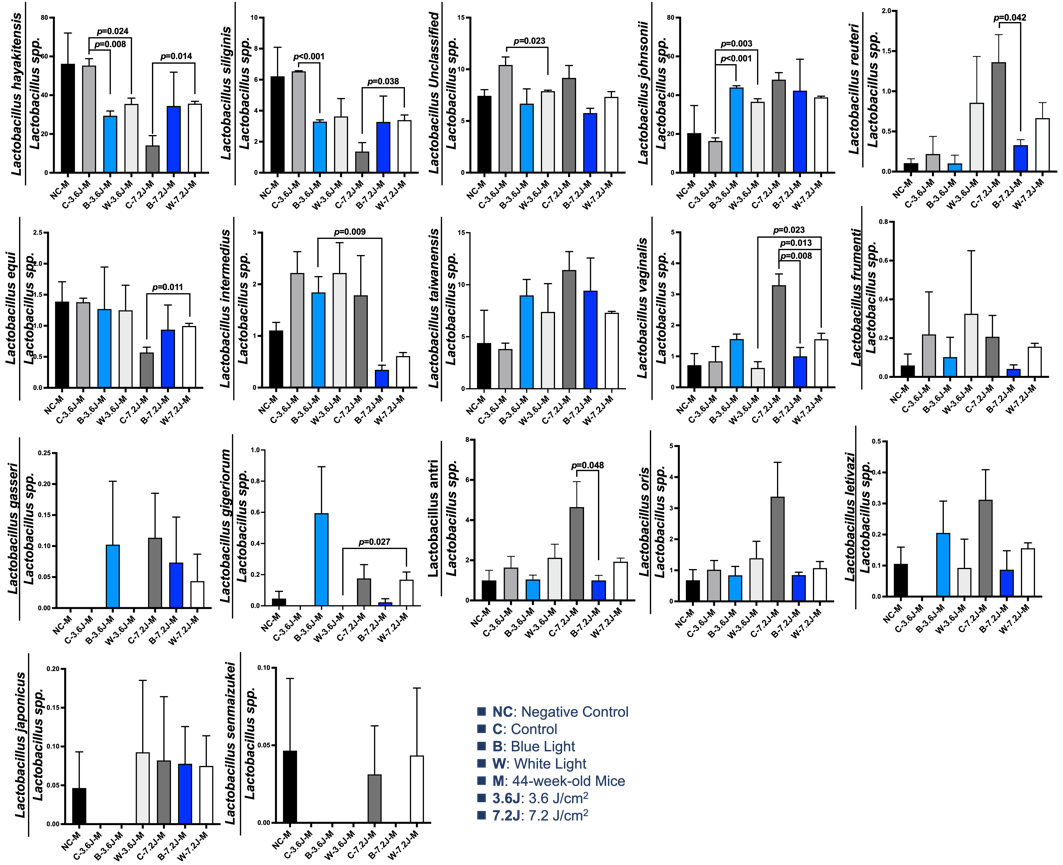
**

**C**

**
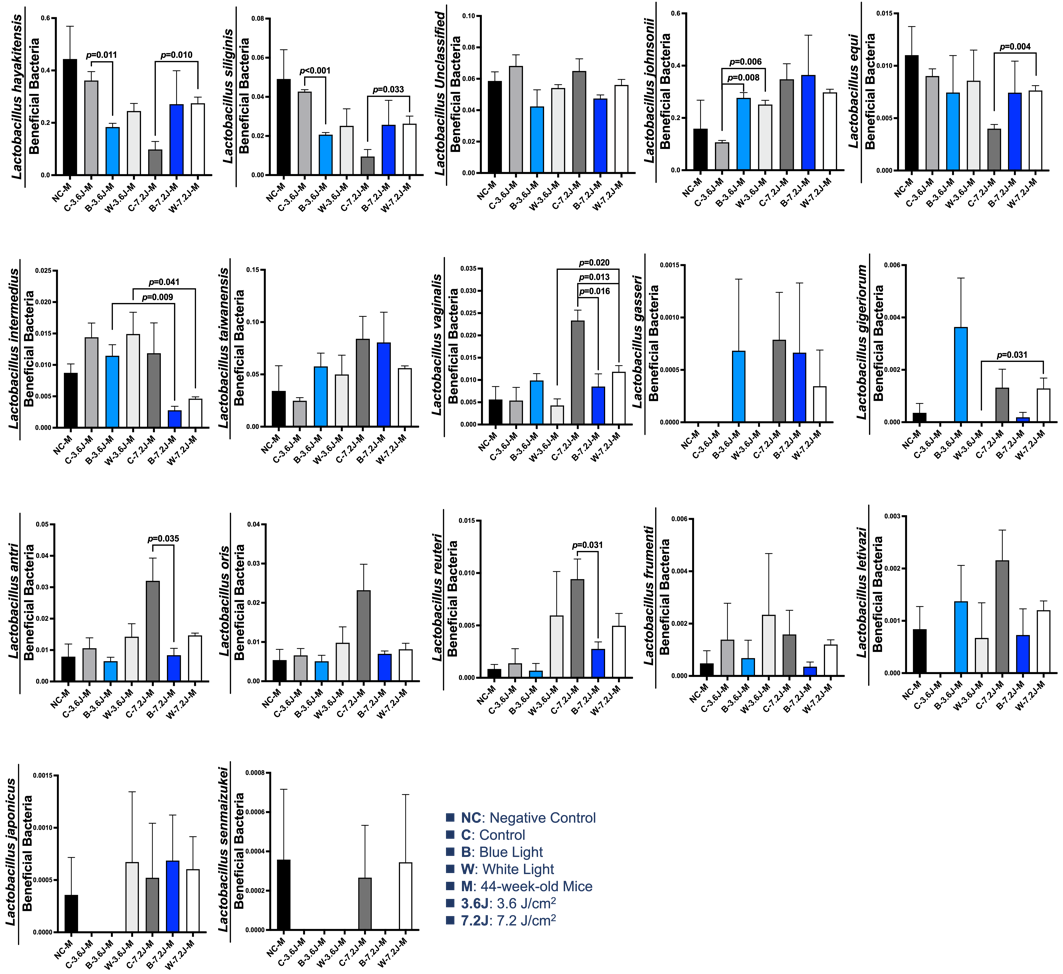
**

**Figure S5. All *Lactobacillus spp.* changed after LED lighting.** (A) *Lactobacillus spp.* were identified by BaseSpace (Illumina). *Lactobacillus hayakitenesis* and *Lactobacillus siliginis* were dramatically increased after a high irradiance of LED lighting. Only *Lactobacillus siliginis* significantly decreased at a low irradiance of blue LED lighting. (B) The bar plot showed the relative abundance of different *Lactobacillus* species in all *Lactobacillus spp.* (C) The bar plot showed the relative abundance of different *Lactobacillus* species in the beneficial bacteria of the cholesterol metabolism and circadian regulation. A *p* value less than 0.05 was considered statistically significant.


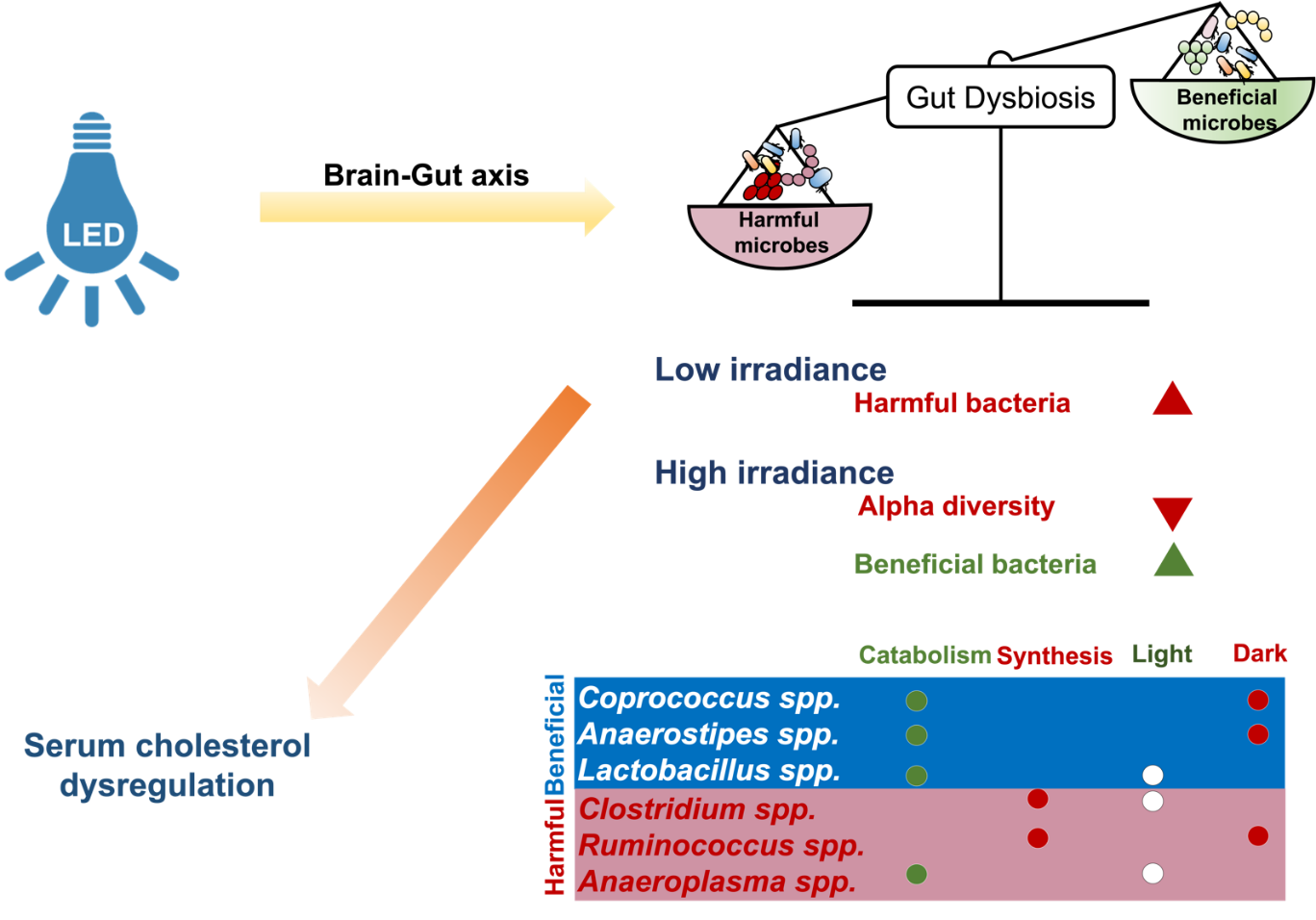


**Figure S6. Schematic summary of the study.** Chronic blue LED lighting might promote gut dysbiosis through the brain-gut axis. At low irradiance, chronic blue LED lighting increased abundance of harmful bacteria, leading to a decreased ratio of beneficial to harmful bacteria, and subsequently elevated serum cholesterol level. At high irradiance, however, blue LED lighting decreased alpha diversity but increased beneficial bacteria as well as the ratio of beneficial to harmful bacteria in turn to reduce serum cholesterol level. The ratio of beneficial to harmful bacteria was negatively correlated with serum cholesterol level but was positively correlated with bile acid biosynthesis. In short, chronic blue LED lighting could harvest gut dysbiosis and specifically dysregulate cholesterol metabolism.
